# Supplementary material for: From sequence to enzyme mechanism using multi-label machine learning
Source: BMC Bioinformatics. 2014 May 19;15:150. doi: 10.1186/1471-2105-15-150 (PMC4229970; doi:10.1186/1471-2105-15-150)
Supplement: Additional file 2 — Java code of ml2db. Additional file ml2db_code.tar.gz contains the Java source code to run the multi-label machine learning experiments and save the results to database. The code’s Javadoc is included. [file 1471-2105-15-150-S2.zip › additional file 2/ml2db/utils/doc/uk/ac/ed/inf/utils/database/class-use/Table.html]

Uses of Class uk.ac.ed.inf.utils.database.Table


---


|  |  |  |
| --- | --- | --- |
| **Overview** \| **Package** \| **Class** \| **Use** \| **Tree** \| **Deprecated** \| **Index** \| **Help** | |  |
| PREV   NEXT | **FRAMES**    **NO FRAMES**     **All Classes** |  |


---


## **Uses of Class uk.ac.ed.inf.utils.database.Table**

| Packages that use Table | |
| --- | --- |
| **test.database** |  |
| **uk.ac.ed.inf.utils.database** |  |

Uses of Table in test.database

| Methods in test.database that return Table | |
| --- | --- |
| `static Table` | `TableTest.getTest1Table()` |

Uses of Table in uk.ac.ed.inf.utils.database

| Methods in uk.ac.ed.inf.utils.database that return Table | |
| --- | --- |
| `Table` | `TableRowSaver.getTable()` |
| `Table` | `TableManager.getTable()` |

| Methods in uk.ac.ed.inf.utils.database with parameters of type Table | |
| --- | --- |
| `TableManager` | `DbManager.getTableManager(Table table)` |

Constructors in uk.ac.ed.inf.utils.database with parameters of type Table

`TableCreator(TableManager tableManager,
Table table)`

`TableManager(DbManager dbManager,
Table table)`
  
          Constructor to create a manager to create, read and write a table.

`TableRowSaver(Table table)`

---


|  |  |  |
| --- | --- | --- |
| **Overview** \| **Package** \| **Class** \| **Use** \| **Tree** \| **Deprecated** \| **Index** \| **Help** | |  |
| PREV   NEXT | **FRAMES**    **NO FRAMES**     **All Classes** |  |


---
